# In Silico Identification and Experimental Validation of (−)-Muqubilin A, a Marine Norterpene Peroxide, as PPARα/γ-RXRα Agonist and RARα Positive Allosteric Modulator

**DOI:** 10.3390/md17020110

**Published:** 2019-02-12

**Authors:** Enrico D’Aniello, Fabio Arturo Iannotti, Lauren G. Falkenberg, Andrea Martella, Alessandra Gentile, Fabrizia De Maio, Maria Letizia Ciavatta, Margherita Gavagnin, Joshua S. Waxman, Vincenzo Di Marzo, Pietro Amodeo, Rosa Maria Vitale

**Affiliations:** 1 Department of Biology and Evolution of Marine Organisms, Stazione Zoologica “Anton Dohrn”, 80121 Naples, Italy; enrico.daniello@szn.it; 2 Endocannabinoid Research Group (ERG), Institute of Biomolecular Chemistry, National Research Council (ICB-CNR), Via Campi Flegrei 34, 80078 Pozzuoli (NA), Italy; fabio.iannotti@icb.cnr.it (F.A.I.); vdimarzo@icb.cnr.it (V.D.M.); 3 Institute of Biomolecular Chemistry, National Research Council (ICB-CNR), Via Campi Flegrei 34, 80078 Pozzuoli (NA), Italy; a.martella@lacdr.leidenuniv.nl (A.M.); alessandra.gentile@mpi-bn.mpg.de (A.G.); fabriziademaio@yahoo.it (F.D.M.); lciavatta@icb.cnr.it (M.L.C.); mgavagnin@icb.cnr.it (M.G.); 4 Molecular Cardiovascular Biology Division, Cincinnati Children’s Hospital Medical Center, Cincinnati, OH 45229, USA; lauren.falkenberg@cchmc.org (L.G.F.); Joshua.Waxman@cchmc.org (J.S.W.); 5 Canada Excellence Research Chair on the Microbiome-Endocannabinoidome Axis in Metabolic Health (CERC-MEND)-Université Laval, Quebec, QC G1V 0A6, Canada

**Keywords:** virtual screening, nuclear receptor agonist, positive allosteric modulator, zebrafish models

## Abstract

The nuclear receptors (NRs) RARα, RXRα, PPARα, and PPARγ represent promising pharmacological targets for the treatment of neurodegenerative diseases. In the search for molecules able to simultaneously target all the above-mentioned NRs, we screened an in-house developed molecular database using a ligand-based approach, identifying (−)-Muqubilin (Muq), a cyclic peroxide norterpene from a marine sponge, as a potential hit. The ability of this compound to stably and effectively bind these NRs was assessed by molecular docking and molecular dynamics simulations. Muq recapitulated all the main interactions of a canonical full agonist for RXRα and both PPARα and PPARγ, whereas the binding mode toward RARα showed peculiar features potentially impairing its activity as full agonist. Luciferase assays confirmed that Muq acts as a full agonist for RXRα, PPARα, and PPARγ with an activity in the low- to sub-micromolar range. On the other hand, in the case of RAR, a very weak agonist activity was observed in the micromolar range. Quite surprisingly, we found that Muq is a positive allosteric modulator for RARα, as both luciferase assays and in vivo analysis using a zebrafish transgenic retinoic acid (RA) reporter line showed that co-administration of Muq with RA produced a potent synergistic enhancement of RARα activation and RA signaling.

## 1. Introduction

Retinoic acid receptors (RARs), retinoid X receptors (RXRs) and peroxisome proliferator-activated receptors (PPARs) belong to the nuclear receptor (NR) family of ligand-activated transcription factors that regulate the expression of genes responsible for several physiological processes including cell growth, differentiation, homeostasis, and apoptosis [1,2]. In mammals, there are three isotypes of each of these NRs, namely, α, β, and γ, which have high sequence homology and different tissue distributions [3,4]. NR family members share a common structural organization comprising an amino-terminal domain (A/B region), a central DNA binding domain (DBD), and a carboxy-terminal ligand-binding domain (LBD). In the absence of ligands, NRs act as transcriptional repressors by inhibiting the expression of downstream target genes. This mechanism is mediated by an interaction with an NR corepressor (NCoR) or the silencing mediator of retinoic acid thyroid hormone receptors (SMRT; also known as NCoR2) together with histone deacetylase 3 (HDAC3) [2]. Ligand binding to the LBD triggers the dissociation of corepressors and the formation of coactivator complexes by altering the conformation of a short helix, termed AF2, in the LBD and the recruitment of specific co-activators such as p300 and members of the steroid receptor coactivator (SRC) subfamily [5]. Once activated by their agonists, the resulting functional hetero- or homodimers bind to the response elements of their target genes, thus initiating transcription. RXRs form functional heterodimers with both RARs and PPARs, but may also form homodimers [1]. PPAR-RXR heterodimers are considered to be “permissive”, meaning that the heterodimers can be activated by either a PPAR or an RXR agonist and, when simultaneously activated, the heterodimer can respond in an additive or synergistic manner [1]. In contrast, RAR-RXR heterodimers are “conditionally permissive”, indicating that they are not activated by RXR ligands alone. Instead, it is the binding of an RAR agonist that initially activates the heterodimers and subsequently allows the binding of RXR ligands to enhance their transcriptional potential [6]. RAR and RXR ligands, referred to as retinoids and rexinoids, respectively, have been used to treat skin disorders and emphysema, as well as in cancer therapies [7]. Moreover, RXRα agonists can reduce fasting plasma glucose and counteract insulin resistance [8], and acting synergistically with PPARγ agonists, are able to improve insulin-sensitivity [9]. Recently, it has also been shown that the stimulation of RARα, RXRα, and PPARα/γ may be beneficial in treating neurodegenerative diseases. In fact, studies carried out with experimental models of Alzheimer’s disease (AD) showed that the treatment with agonists of these NRs improved cognition and memory, improving disease-related pathology. Specifically, RXR agonists can stimulate the physiological clearance of amyloid-β (Aβ) [10], whereas PPARγ agonists suppress the Aβ-mediated activation of microglia in vitro and prevent neural cell death [11,12] and PPARα agonists can reduce endogenous Aβ production by inducing the α-secretase-mediated proteolysis of amyloid precursor protein (APP) [13]. Furthermore, it was found that dietary deficiency of vitamin A disrupts the retinoid signaling pathway in adult rats, leading to the deposition of amyloid-β in the cerebral blood vessels via down-regulation of RARα in forebrain neurons and the loss of choline acetyltransferase (ChAT) expression [14,15]. These changes were reversed by the administration of retinoic acid (RA). Moreover, histological samples from AD patients showed a deficit of RARα expression and a deposition of Aβ in the surviving neurons [14]. Altogether, the ability of RAR and PPAR stimulation to affect Aβ accumulation suggests that compounds with broad agonist activity toward RARα, RXRα, and PPAR-α and -γ could represent useful pharmacological tools to provide novel inroads for the treatment of neurodegenerative diseases. 

Natural compounds represent an invaluable source of bioactive compounds potentially able to act on multiple-related targets due to their versatile scaffolds and functional groups. By continuing our search for multiligand agents from natural sources [16,17,18], we focused our attention in this work on compounds potentially able to bind and activate nuclear receptors such as RXRα, RARα, and PPARα/γ, because they may have therapeutical potential for the treatment of neurological diseases such as Alzheimer’s disease. With this aim, we used a ligand-based approach [19] to screen and identify possible candidates in our in house-developed database StOrMoDB (Structurally Oriented Molecular DataBase) of marine natural compounds and found (−)-Muqubilin A (Muq) as a potential hit. Muq is a norterpene cyclic peroxide described from distinct Red Sea sponges [20,21] and recently isolated in our institute from the sponge *Diacarnus erythraeanus* [22]. Norterpene cyclic peroxides are active metabolites endowed with cytoxic activity against a variety of cancer cell lines [21,22,23,24]. The ability of this compound to act as a potential multiligand agent for RARα-RXRα-PPARα/γ was assessed by molecular docking and molecular dynamics simulations and then validated by luciferase assays. While Muq was found to act as a full agonist of RXRα, PPARα, and PPARγ, and as a weak agonist for RARα, luciferase and in vivo transgenic reporters in zebrafish showed that it is also a positive allosteric modulator of RARα. Therefore, our results indicated that the marine compound Muq is endowed with a broad agonist activity for multiple NRs, which may suggest its potential use against neurodegenerative diseases.

## 2. Results

### 2.1. Virtual Screening of Our In-House Molecular Database 

**Figure 1:**
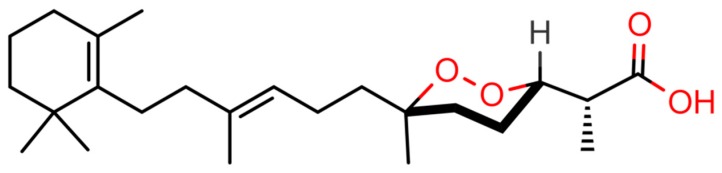
Chemical structure of (-)-Muqubilin (Muq) A. Heteroatoms and polar hydrogens are colored red.

A ligand-based virtual screening (VS) approach was used to identify possible RXRα, RARα, and PPARα/γ multiligands in our in house-developed database StOrMoDB, which contains 350 compounds of marine origin, isolated and characterized at the Institute of Biomolecular Chemistry, National Research Council (ICB-CNR), for which samples are currently available for experimental validation. The VS was performed by running DB queries based on SMARTS strings (see Section 4.1, “Computational Methods”) encoding the RA-derived scaffold that we hypothesized to provide multiligand properties against the selected targets. This scaffold featured a 2,6,6-trimethylcyclohexen-1-yl ring (TMCH), that is, the pendant group of RA, and a carboxylate group, separated by a proper spacer producing an elongated structure, which can ensure a favorable ligand accommodation in the LBD of both retinoids and PPAR proteins. The search produced a norterpene cyclic peroxide (−)-Muqubilin A, here termed Muq, as a positive hit (Figure 1). Muq was then subjected to molecular docking and molecular dynamics (MD), as described in the experimental section, to assess the propensity of the molecule to effectively bind the above-mentioned molecular targets. 

### 2.2. Muq-RXRα Complex Model

**Figure 2:**
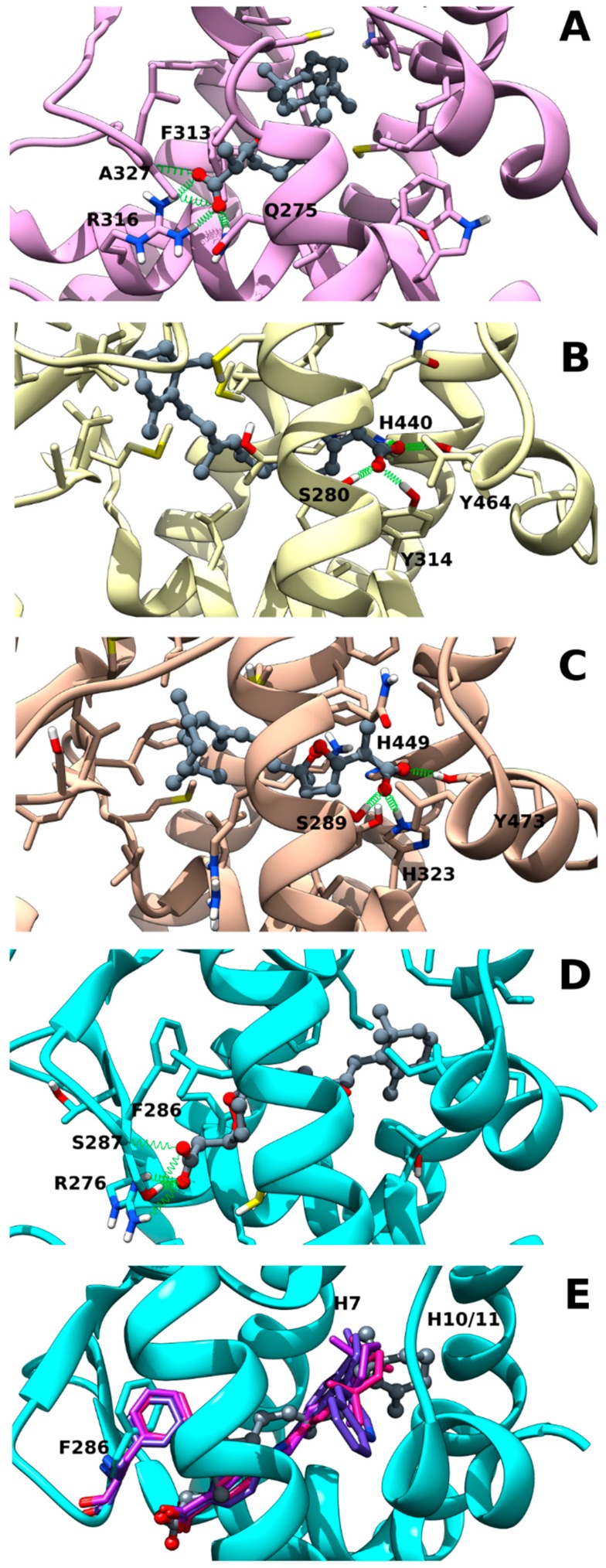
Representative frames from molecular dynamics (MD) of Muq-nuclear receptor (NR) complexes. (**A**) Muq-RXRα; (**B**) Muq-PPARα; (**C**) Muq-PPARγ; (**D**) Muq-RARα; (**E**) best fit between Muq-RARα MD frame (protein and Muq shown) and experimental structures of RARα in complex with agonists (only ligands shown: PDB ID: 3KMR, magenta; 3A9E, purple; 1DKF, blue). Ball and stick and stick-only representations are used for ligand heavy atoms and protein sidechain atoms within 5 Å from ligand, respectively. Carbon atoms are painted according to ribbons for proteins and dark gray for Muq, Hydrogen, nitrogen, oxygen, and sulfur atoms are painted white, blue, red, and yellow, respectively. A “green spring” representation is adopted for H-bonds involving ligand atoms.

The best docking pose of Muq into RXRα underwent 50 ns of MD to assess its stability. Analysis of the MD trajectory (Figure S2, Supplementary Materials) shows that Muq adopts a very stable pose within the LBD, preserving an arrangement similar to the starting docking pose. Figure 2A illustrates the last MD frame as representative of this complex. The molecule is hosted in the hydrophobic pocket formed by residues lying on helices H3, H5, H7, and H11 and on the β-turn. The carboxylate group is involved in three H-bond interactions, a polar network also found in the experimental *cis*-retinoic acid-RXRα complex structure (PDB ID: 1FBY): one reinforced by ionic interaction with Arg316(H5) (MD occurrence >90%), and the others with Gln275(H3) side-chain (MD occurrence >55%) and with the amide backbone of Ala327(β-turn) (MD occurrence >58%). The cyclic peroxide is sandwiched between Phe313(H5) and Ala 272(H3), whereas the cyclohexenyl ring is hosted in the hydrophobic pocket formed by helices H7 and H11, which is the same site docking the TMCH group in the experimental *cis*-retinoic acid-RXRα complex structure. The overall arrangement of Muq in the LBD and the network of polar interactions engaged by the molecule is strongly suggestive of a full agonism behavior on RXRα.

### 2.3. Muq-PPARα/γ Complex Models

The same computational protocol adopted for RXRα was also applied to PPARα and PPARγ molecular targets. The resulting complexes show that Muq is well accommodated in the LBD of both PPARs, giving rise to stable complexes, which exhibit neither significant drift throughout the whole MD simulations nor appreciable deviations from their starting docking poses. However, the formation of interactions with residues lying on the Ω-loop during MD induces a more extended ligand conformation in the PPARα-Muq complex in comparison with the starting docking pose. On the other hand, the Muq pose in the PPARγ complex, even if characterized by a higher flexibility in comparison to PPARα (see Figure S2, Supplementary Materials), preserves in MD the same overall arrangement of the starting docking pose. The last frames of each complex, taken as representative of the corresponding trajectories, are shown in Figure 2B,C. Muq adopts a horseshoe conformation embracing helix H3 in both PPAR binding sites, orienting its carboxyl group toward H12 and the cyclohexenyl ring toward the β-sheet and the Ω loop. The carboxyl group engages a network of stable H-bond interactions with PPARα-Tyr464 (MD occurrence >60%)/PPARγ-Tyr473(H12) (MD occurrence >90%), PPARα-His440 (MD occurrence >55%)/PPARγ-His449(H10/11) (MD occurrence >38%), PPARα-Tyr314 (MD occurrence >80%)/PPARγ-His323(H5) (MD occurrence >55%), and PPARα-Ser280(MD occurrence >11%)/PPARγ-Ser289(H3) (MD occurrence >21%). Since Muq exhibits overall arrangements in both PPAR isoforms that recapitulate those of classical PPARα/γ full agonists, it is expected to act as a dual PPARα/γ agonist.

### 2.4. Muq-RARα Complex Model

The in silico screening was also used to evaluate the potential ability of Muq to bind RARα. As with the other NRs, Muq is able to accommodate well into the LBD, adopting a binding stable pose during the whole MD simulation, even if characterized by higher flexibility than in the RXRα complex (see Figure S2, Supplementary Materials) The last frame from MD was taken as representative and is shown in Figure 2D. Ligand-protein H-bonds are formed between the Muq carboxylate group and both Ser287(β-turn) backbone/sidechain (MD occurrence >50% and 90%, respectively) and Arg276 (H5) sidechain (MD occurrence >75%), this latter reinforced by ionic interactions. This polar network is common to known agonists in their experimental structures of RARα complexes (PDB ID: 3KMR, 3A9E, and 1DKF). However, the overall arrangement of Muq in the RARα LBD shows peculiar features since the methyl on the peroxide ring induces a rotation of Phe286(β-turn) sidechain and the cyclohexenyl ring occupies a larger fraction of cavity between helices H7 and H10/11 in comparison with other agonists in their crystallographic complexes with RARα (Figure 2E). These features could in principle negatively affect the ability of Muq to act as a full agonist for this NR.

### 2.5. Muq is an Agonist for RXRα and PPARα/γ

To confirm the computational predictions of Muq binding to RXRα receptors, we transfected COS-7 cells with human chimeric RXRα-LBD-Gal4 constructs together with the TK-MH100x4 construct, which contain four direct tandem copies of the UAS (upstream activating sequence) enhancer. These types of fusion proteins have been used to assess the functions of individual NR domains [25]. The day after plating, cells were treated with the RXR agonist 9-*cis*-retinoic acid and Muq at 0.1, 1, and 10 µM. Higher concentrations were not investigated since in a separate set of experiments, COS-7 cell viability measured by MTT assays was significantly reduced (see Figure S1, Supplementary Materials), in agreement with a previous study reporting a reduction of viability with a mean IC_50_ of 17 ± 7 µM and 9 ± 2 µM in normal (fibroblasts, epithelial cells, and keratinocytes) and cancer cell lines, respectively [22].

**Figure 3:**
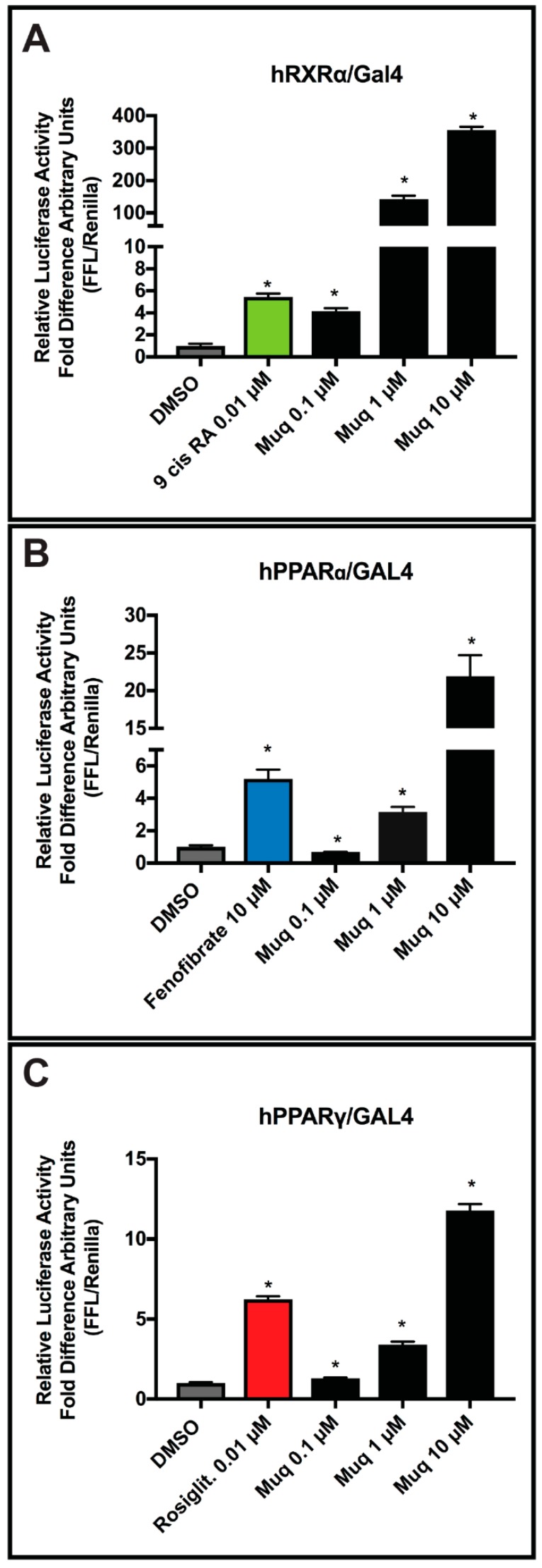
Luciferase assays. (**A**) Muq acts as an agonist on hRXRα-LBD. Relative luciferase activity in response to 0.1, 1, and 10 µM of Muq. 9-*cis*-retinoic acid at 0.01 µM was used as an RXRα agonist positive control. (**B**) Muq acts as an agonist on hPPARα-LBD. Relative luciferase units in response to 0.1, 1, and 10 µM of Muq. Fenofibrate at 10 µM was used as an hPPARα agonist positive control. (**C**) Muq acts as an agonist on hPPARγ-LBD. Relative luciferase units in response to 0.1, 1, and 10 µM of Muq. Rosiglitazone at 0.01 µM was used as an hPPARγ agonist positive control. The activity of the vehicle control was set at 1 and the relative luciferase activity obtained for each control and concentrations tested are presented as a fold induction with respect to the vehicle control. Statistical analysis was performed by comparing each concentration of Muq and of the specific agonist to the vehicle control using the Student’s *t*-test. The analyses were performed using GraphPad Prism 7 software. Statistically significant differences were accepted when the *p*-value was at least ≤0.05. Data are expressed as means ± SEM, (*n* = 3). * *p*  ≤  0.05.

Results in Figure 3A clearly show that in vitro Muq is able to activate the *h*RXRα fusion protein as a full agonist in a concentration–response manner, in agreement with the computational data, with a 10 µM concentration promoting a >300-fold increase in measured activation. Thus, Muq can function as a strong agonist for *h*RXRα. Given these results, we posit that the activity of Muq as a potent RXR agonist could also explain the greater cytotoxicity exhibited by this molecule toward the cancer cell lines in comparison with the normal ones [22], since RXR agonists are known to act as effective chemotherapeutic agents [26,27]. 

Next, we examined if Muq was able to function as an agonist for *h*PPARα/γ receptors as predicted by our computational study. As with the *h*RXRα-LBD-Gal4 fusion, the ability of Muq to activate these receptors was assayed using chimeric *h*PPARα-LBD-Gal4 and *h*PPARγ-LBD-Gal4 constructs transfected together with a UAS enhancer. As shown in Figure 3B,C, Muq was able to induce transcriptional activation of both PPAR-Gal4 chimeras, but produced a greater activation by *h*PPARα than the *h*PPARγ fusion. Overall, our in vitro data confirm the full agonist ability of Muq for both *h*PPARα and *h*PPARγ receptors.

### 2.6. Muq Functions as an Allosteric Enhancer of RA on hRARα

**Figure 4:**
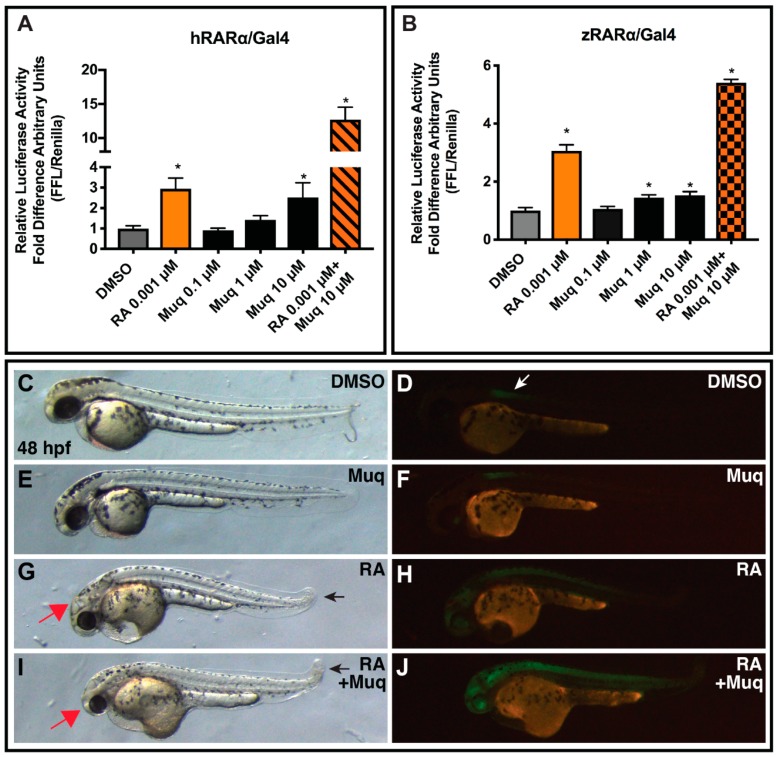
Muq synergistically reinforces (**A**) human RARα in vitro and (**B**) zebrafish RARα in vitro. Relative luciferase units in response to 0.1, 1, and 10 µM of Muq. Retinoic acid (RA) at 0.001 µM was used as an *h*RARα and a zRARα agonist positive control. The activity of the vehicle control was set at 1 and the relative luciferase activities obtained for each control and concentration tested are presented as a fold induction with respect to the vehicle control. Statistical analysis was performed by comparing each concentration of Muq and that of RA to the vehicle control using the Student’s *t*-test. The analyses were performed using GraphPad Prism 7 software. Statistically significant differences were accepted when the *p*-value was at least ≤0.05. Data are expressed as means ± SEM, (n = 9) for human RARα and (*n* = 3) for zebrafish RARα. * *p*  ≤  0.05. (**C**) Muq behaves as a weak RARα agonist and enhances RA signaling in zebrafish. Embryos carrying the *12XRARE-ef1a:EGFP* transgene at 48 hpf that were treated from 24 hpf with (**C**,**D**) DMSO, (**E**,**F**) 10 μM Muq, (**G**,**H**) 0.5 μM RA, and (**I**,**J**) 0.5 μM RA + 10 μM Muq. Embryos in **C**, **E**, **G**, and **I** are the same as **D**, **F**, **H**, and **J**. Red arrows in **G** and **I** indicate smaller eyes. Black arrows in **G** and **I** indicate shortened tail. White arrow indicates E-GFP from the transgene in the anterior spinal cord. For this study, 25 embryos per condition were examined.

While our computational results suggested a canonical agonist behavior for Muq on RXRα and PPARα/γ, the results for RARα, although still supporting an appreciable interaction with the receptor, showed a significant deviation from structural requirements for canonical agonism. By using *h*RARα-LBD-Gal4 fusion proteins, again equivalent to those assayed for the other NRs, we found that Muq weakly stimulated *h*RARα-LBD-Gal4 proteins compared to RA in the reporter assay (Figure 4A). However, unexpectedly, we found that the co-administration of RA with Muq showed a synergistic enhancement of reporter activation that cannot be ascribed to the agonistic effect of Muq on RXRα, since the Gal4-fusion construct should function independently from RXRα. Given these surprising results obtained analyzing the *h*RARα-LDB fusion, we sought to confirm the synergistic effect of Muq with RA by carrying out additional in vitro and in vivo experiments. We chose zebrafish embryos due to the accessibility of available tools to assess RAR function and RA signaling in these animals [28]. Moreover, RARs are highly conserved proteins in vertebrates [29], with the LBD of zebrafish RARαa/b sharing >90% sequence identity with the LBD of *h*RARα. We first performed the UAS luciferase reporter assays using a zebrafish RARαb-LBD-Gal4 chimeric construct [30]. We obtained similar results in comparison with the *h*RARα-LBD-Gal4 fusion, again observing a weak activation alone and a potentiating effect of Muq with RA (Figure 4B). Together, the in vitro analysis strongly suggests the presence of an additional, positive allosteric site for Muq, since the orthosteric one would not allow the simultaneous binding of both Muq and RA. 

Finally, we wanted to determine if Muq was able to act as enhancer of RA also in vivo. Therefore, we treated zebrafish embryos carrying the RA signaling reporter line *Tg(12XRARE-elf1a:EGFP)^sk^*^72^ [31] from 24 hpf until 48 hpf with 0.5 µM RA and 10 µM Muq alone and together, and then we analyzed the morphology of the embryos and the expression of GFP. A 0.5 µM portion of RA was used, in contrast to 0.001 µM RA from the in vitro reporters, because this produces a perceptible, yet minimal upregulation of GFP in vivo (Figure 4F). While 10 µM Muq did not produce a perceptible effect on the embryos compared to the DMSO (control) treatment (Figure 4F), the combination of RA and Muq enhanced both the teratogenic morphological defects (smaller heads and shortened tails [28,29]) and the GFP expression of the RA reporter compared to RA treatment alone (Figure 4I,J). Therefore, our in vivo analysis of Muq with RA in zebrafish embryos recapitulates the same trends observed in the in vitro assays using chimeric humans and zebrafish RARα.

## 3. Discussion and Conclusions

The norterpene cyclic peroxide Muq emerging from the search in our in house-developed database StOrMoDB was subjected to molecular docking and molecular dynamics simulations to assess its ability to actually bind the aforementioned NRs. In fact, this class of molecules has been previously reported as cytotoxic [21,22] and/or antimalarial agents [32], but not as NR ligands. The binding modes found for Muq in the LBD of RXRα, PPARα, and PPARγ were compatible with a full agonism, since both the pattern of the polar interactions and the orientation of the hydrophobic tail recapitulated the binding mode of canonical agonists. In fact, full PPAR agonists adopt a horse-shoe conformation in the LBD, engaging H-bonds with Ser(H3)/His(H10/119) and Tyr(H12), and hydrophobic interactions with residues lying on the β-sheet, whereas RXR agonists engage H-bonds involving Arg316(H5) sidechain and Ala (β-turn) backbone with the hydrophobic pendant lying between helices H7 and H10/11. On the other hand, for RARα LBD, the binding mode of Muq showed some peculiar features in comparison with other experimentally solved complexes with agonists, potentially impairing the ability of this compound to act as a canonical agonist. In particular, the TMCH group of Muq occupies a larger volume of the pocket between helices H7 and H10/11, and the cyclic peroxide group induces a rotation of the β-turn Phe286 sidechain, whose orientation is well conserved in the experimental complexes. The subsequent validation of the computational results by luciferase assays confirmed that Muq is a potent *h*RXRα agonist and a full agonist for *h*PPARα/γ, whereas it is a only a weak agonist for *h*RARα. Thus, the peculiar features observed in the binding mode of Muq into the RARα LBD negatively affected its ability to directly activate this NR. However, since MD predicted a stable binding for the Muq-RARα complex, we also evaluated the ability of Muq to modulate the effect of RA. The co-administration of Muq and RA showed a strong additive effect, which can be neither ascribed to the effects of Muq on *h*RXRα, since the Gal4 construct was used in luciferase assays, nor predicted from the modelled structure of the Muq-RARα complex shown in Figure 2E, which would not allow the coexistence of Muq and RA in the LBD site. This interesting finding was confirmed by both luciferase assays on zebrafish RXRαb LBD and morphological and GFP-expression studies on zebrafish embryos, disclosing an effect of Muq as positive allosteric modulator for this NR. 

In conclusion, using a combination of computational and experimental approaches, we have identified Muq as a novel multiligand exerting full agonist activity at RXRα and PPARα/γ receptors and positive allosteric modulatory activity at RARα. Importantly, our results propose Muq as a candidate in the treatment of neurodegenerative diseases, such as Alzheimer’s disease, where a broad stimulation of this NR pool could provide greater benefits than pharmacological approaches targeting each NR individually. Moreover, the potent agonism of Muq at RXRα could also explain the previously reported cytotoxic activity exhibited against different cancer cell lines [22]. To the best of our knowledge, Muq represents the first example of a positive allosteric modulator of RARα. Although further studies are required to identify the allosteric binding site of RARα, and to assess the activity of Muq in experimental models of disease, we envision that our results could pave the way to a deeper understanding of the mechanisms regulating NR function and to the discovery of new pharmacological tools to treat neurological diseases and cancers.

## 4. Materials and Methods 

### 4.1. Computational Methods

Virtual screening of the StOrMoDB database (https://stormodb.na.icb.cnr.it/stormodb) was performed using the featured SMARTS pattern querying option on DB compounds for which samples for experimental validation were eventually available (350 molecules, all of marine origin). To search for molecules featuring a 2,6,6-trimethylcyclohexen-1-yl ring (TMCH) separated from a carboxylate group by a variable-length spacer, the general SMARTS pattern CC1(C)CCCC(C)=C1C-,=,#,@{[*]-,=,#,@}n[C,c](=[O,o])[OH,oH,O-,o-] (where “{[*]-,=,#,@}n“ stands for “n repeats of the [*]-,=,#,@ pattern”) was used in fourteen queries in which the spacer length n was allowed to vary from 3 to 16. The only hit, Muq, was obtained for n = 10.

Starting ligand geometry was built with Ghemical 2.99.2 [33] followed by energy minimization (EM) at molecular mechanics level first, using Tripos 5.2 force field parametrization [34], and then at AM1 semi-empirical level; fully optimized using GAMESS program [35] at the Hartree–Fock level with STO-3G basis set; and subjected to HF/6-31G^*^/STO-3G single-point calculations to derive the partial atomic charges by the RESP (Restrained ElectroStatic Potential) procedure [36]. Docking studies were performed with AutoDock Vina1.1.2 [37], by using the crystallographic structures of RXRα, RARα, PPARα, and PPARγ (PDB:3R5M, 3KMR, 2P54, and 2F4B, respectively). Both proteins and ligands were processed with the AutoDock Tools (ADT) package version 1.5.6rc1 [38] to merge non polar hydrogens, calculate Gasteiger charges, and select the rotatable side-chain bonds. Grids for docking evaluation were generated using the program AutoGrid 4.2 included in AutoDock 4.2 distribution, setting a spacing of 0.375 Å and a resolution of 60 × 60 × 60 points for PPARγ and RXRα, 50 × 50 × 70 points for PPARα, and 60 × 60 × 70 points for RARα, centered on the ligand-binding site, by positioning the center of the grid in proximity of Tyr327 for PPARγ, Ser280 for PPARα, Cys432 for RXRα, and Leu269 for RARα. The following parameters were used for AutoDock Vina: exhaustiveness of 8, energy threshold of 10 kcal mol^−1^, and up to 100 poses for each compound (Vina automatically only reports “interesting” poses within the energy threshold, which, for the selected input parameters, produced 20 poses per run for all the docking runs with the exception of those for the X-ray ligands of PPARs, which were four for PPARα and two for PPARγ). For each simulated complex, the pose exhibiting the most favorable Vina binding energy value was selected as the representative binding pose from docking. For comparison purposes, reference compounds were used in docking calculations: RA for both RXRα and RARα and the corresponding X-ray ligands for PPARα and PPARγ. The energy values for the best-scoring poses between Muq and the reference compounds were comparable. In detail, we obtained values of −10.0 (RA) vs. −8.2 kcal mol^−1^ (Muq) for RXRα, −9.1 (RA) vs. −10.0 kcal mol^−1^ (Muq) for RARα, −6.9 (GW735) vs. −6.8 kcal mol^−1^ (Muq) for PPARα, and −12.6 (((5-{3-[(6-benzoyl-1-propyl-2-naphthyl)oxy]propoxy}-1H-indol-1-yl) acetic acid) and −8.2 (Muq) kcal mol^−1^ for PPARγ. The selected complexes were then completed by the addition of all hydrogen atoms and underwent EM and then MD simulations with Amber16 pmemd.cuda module [39], using the ff14SB version of AMBER force field for the protein and gaff parameters [40] for the ligand. 

To perform MD simulations in solvent, the complexes were confined in TIP3P water periodic truncated octahedron boxes exhibiting a minimum distance between solute atoms and box surfaces of 10 Å, using the tleap module of the AmberTools16 package. The systems were then neutralized by the addition of counterions (Na^+^) and subjected to 1000 steps of EM with solute atoms harmonically restrained to their starting positions (K_r_ = 10 kcal mol^−1^ Å^−1^). Then, 90 ps restrained MD (K_r_ = 5 kcal mol^−1^ Å^−1^) at constant volume was run on each solvated complex, gradually heating the system to 300 K, followed by 60 ps restrained MD (K_r_ = 5 kcal mol^−1^ Å^−1^) at constant temperature (300 K) and pressure (1 atm) to adjust system density. Production MD simulations were carried out at constant temperature (300 K) and pressure (1 atm) for 50–100 ns, with a time-step of 2 fs. Bonds involving hydrogens were constrained using the SHAKE algorithm [41]. The Cpptraj module of AmberTools16 and program UCSF Chimera 1.10.1 [42] were used to perform MD analysis and to draw the figures, respectively.

### 4.2. Purification of (−)-Muqubilin A

The amount of Muq used in tests was obtained from the lipophilic extract of the Red Sea sponge *Diacarnus erythraeanus*. Isolation, purification, and identification of Muq have been previously described [22].

### 4.3. Luciferase Assay

COS-7 cells (monkey kidney fibroblast-like cells) were grown in DMEM supplemented with 10% fetal bovine serum and 1% Pen/Strep under standard conditions. Cells were plated in 24-well dishes at 70% confluence and transfected using Lipofectamine LTX and PLUS Reagent (cat. 15338-100; Thermo Fisher Scientific, Milan IT) according to the manufacturer’s instructions. 

Briefly, at day 1, for each well, a combination of 25 ng of CMX-Gal4-*h*RXRα, or 25 ng of pM1-*h*PPARα-Gal4, or pM1-*h*PPARγ-Gal4, or pSG5-Gal4-*h*RARα, or with zebrafish RARα was transfected together with 300 ng of TK-MH100x4-Luc containing the UAS enhancer elements, 25 ng of Renilla Luciferase (pRL, cat. E2231; Promega, Milan, Italy), and 150 ng pcDNA3 (Thermo Fisher Scientific, Milan, Italy) empty vector to obtain a total of 500 ng. The next day, the growth media was replaced with fresh media containing compounds of interest. The DMSO was used as vehicle. Fenofibrate (cat. F6020; Sigma-Aldrich), rosiglitazone (cat. 5325; Tocris, Bristol, UK), 9-*cis*-retinoic acid (cat. R4643; Sigma-Aldrich), and retinoic acid (cat. R2625; Sigma-Aldrich) were used respectively as agonist of PPARα, PPARγ, hRXRα, and human and zebrafish RARα. On day 3, after 18 hours of treatment, the cells were harvested and processed for the luciferase and Renilla luciferase (Promega) using the Dual-Luciferase Reporter Assay System (Promega, cat. E1910) and detected using the GloMax Luminometer (Promega). 

### 4.4. MTT Assay

COS-7 cells were grown in DMEM supplemented with 10% FBS, l-glutamine and 1% Pen/Strep under standard conditions in 48-well cell culture plates. After adhesion, cells were serum-deprived and treated with the desired concentrations of compounds for 18 h (the absence of serum was maintained during the treatments). Cell viability was assessed by the MTT assay following published procedures [43].

### 4.5. Zebrafish Husbandry and Treatments

All zebrafish husbandry and experiments were performed in accordance with protocols approved by the Institutional Animal Care and Use Committee (IACUC) of Cincinnati Children’s Hospital Medical Center. The zebrafish transgenic line *Tg(12XRARE-elf1a:EGFP)^sk^*^72^ was used in experiments. Zebrafish embryo were treated with RA and/or Muq at the concentrations indicated above. Then, 25 embryos per condition were placed in glass vials with 2 mL of embryo water. RA from a 10^−1^ M stock in DMSO was diluted 1:100 in DMSO followed by a subsequent dilution to the final concentration in fish water. Muq was diluted to the final concentration in embryo water from a 10^−2^ M stock in DMSO. Embryos were placed on a nutator in an incubator for 24 hpf. Their morphology and fluorescence were examined and imaged using a Zeiss M2Bio_V8 (Carl Zeiss Microscopy, Thornwood, NY, USA) fluorescent microscope.

## Supplementary Materials

The following are available online at http://www.mdpi.com/1660-3397/17/2/110/s1, Figure S1: Effect of Muqubilin on the viability of COS-7 cells, Figure S2: Evaluation of stability of the ligand binding mode during the last 30 ns of MD.
