# Supplementary material for: In Silico Identification and Experimental Validation of (−)-Muqubilin A, a Marine Norterpene Peroxide, as PPARα/γ-RXRα Agonist and RARα Positive Allosteric Modulator
Source: Mar Drugs. 2019 Feb 12;17(2):110. doi: 10.3390/md17020110 (PMC6410278; doi:10.3390/md17020110)
Supplement: Supplementary file 1 [file marinedrugs-17-00110-s001.pdf]

Supplementary Material for

In silico identification and experimental validation of (−)-Muqubilin A, a marine norterpene peroxide, as PPARα/γ-RXRα agonist and RARα positive allosteric modulator.

Enrico D'Aniello[1,2], Fabio Arturo Iannotti[3,4], Lauren G. Falkenberg[5], Andrea Martella[6], Alessandra Gentile[7], Fabrizia De Maio[8], Maria Letizia Ciavatta[9], Margherita Gavagnin[10], Joshua S. Waxman[11], Vincenzo Di Marzo[12,13,14], Pietro Amodeo[15*] and Rosa Maria Vitale[16*]

1 Department of Biology and Evolution of Marine Organisms, Stazione Zoologica "Anton Dohrn", 80121, Naples, Italy; enrico.daniello@szn.it

2 Endocannabinoid Research Group (ERG), Institute of Biomolecular Chemistry, National Research Council (ICB-CNR), Via Campi Flegrei 34, 80078, Pozzuoli (NA), Italy; enrico.daniello@szn.it

3 Endocannabinoid Research Group (ERG), Institute of Biomolecular Chemistry, National Research Council (ICB-CNR), Via Campi Flegrei 34, 80078, Pozzuoli (NA), Italy; fabio.iannotti@icb.cnr.it

4 Institute of Biomolecular Chemistry, National Research Council (ICB-CNR), Via Campi Flegrei 34, 80078, Pozzuoli, (NA), Italy; fabio.iannotti@icb.cnr.it

5 Molecular Cardiovascular Biology Division, Cincinnati Children's Hospital Medical Center, Cincinnati, OH 45229, USA; lauren.falkenberg@cchmc.org

6 Institute of Biomolecular Chemistry, National Research Council (ICB-CNR), Via Campi Flegrei 34, 80078, Pozzuoli, (NA), Italy; a.martella@lacdr.leidenuniv.nl

7 Institute of Biomolecular Chemistry, National Research Council (ICB-CNR), Via Campi Flegrei 34, 80078, Pozzuoli, (NA), Italy; alessandra.gentile@mpi-bn.mpg.de

8 Institute of Biomolecular Chemistry, National Research Council (ICB-CNR), Via Campi Flegrei 34, 80078, Pozzuoli, (NA), Italy; fabriziademaio@yahoo.it

9 Institute of Biomolecular Chemistry, National Research Council (ICB-CNR), Via Campi Flegrei 34, 80078, Pozzuoli, (NA), Italy; lciavatta@icb.cnr.it

10 Institute of Biomolecular Chemistry, National Research Council (ICB-CNR), Via Campi Flegrei 34, 80078, Pozzuoli, (NA), Italy; mgavagnin@icb.cnr.it

11 Molecular Cardiovascular Biology Division, Cincinnati Children's Hospital Medical Center, Cincinnati, OH 45229, USA; Joshua.Waxman@cchmc.org

12 Institute of Biomolecular Chemistry, National Research Council (ICB-CNR), Via Campi Flegrei 34, 80078, Pozzuoli, (NA), Italy; vdimarzo@icb.cnr.it

13 Endocannabinoid Research Group (ERG), Institute of Biomolecular Chemistry, National Research Council (ICB-CNR), Via Campi Flegrei 34, 80078, Pozzuoli (NA); vdimarzo@icb.cnr.it

14 Canada Excellence Research Chair on the Microbiome-Endocannabinoidome Axis in Metabolic Health (CERC-MEND)-Université Laval, Quebec City, Canada; vdimarzo@icb.cnr.it

15 Institute of Biomolecular Chemistry, National Research Council (ICB-CNR), Via Campi Flegrei 34, 80078, Pozzuoli, (NA), Italy; pamodeo@icb.cnr.it

16 Institute of Biomolecular Chemistry, National Research Council (ICB-CNR), Via Campi Flegrei 34, 80078, Pozzuoli, (NA), Italy; rmvitale@icb.cnr.it

* Correspondence: pamodeo@icb.cnr.it, rmvitale@icb.cnr.it

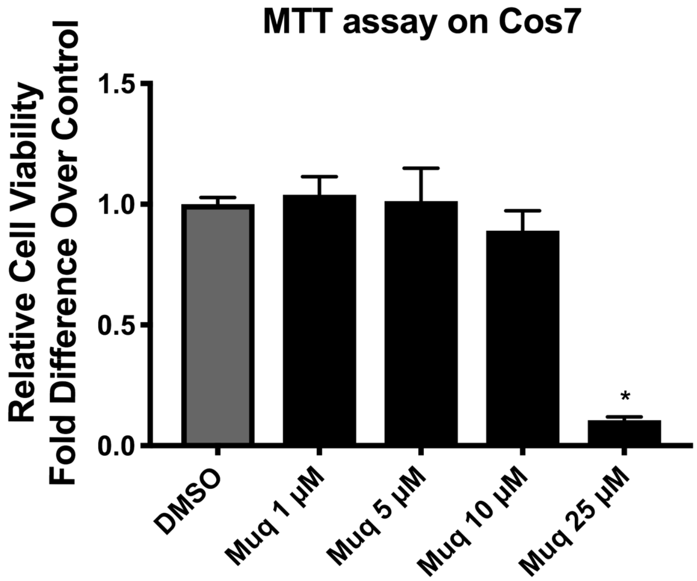

S1: Effect of Muqubilin on the viability of COS-7 cells.
Cell viability was assessed using the MTT assay. Statistical analysis was performed comparing each compound to the DMSO group using the Student *t-test*. Statistically significant differences were accepted when the p-value was at least ≤ 0.05. Data are expressed as means ± SEM, (n=3). $^{*}p \leq 0.05$.

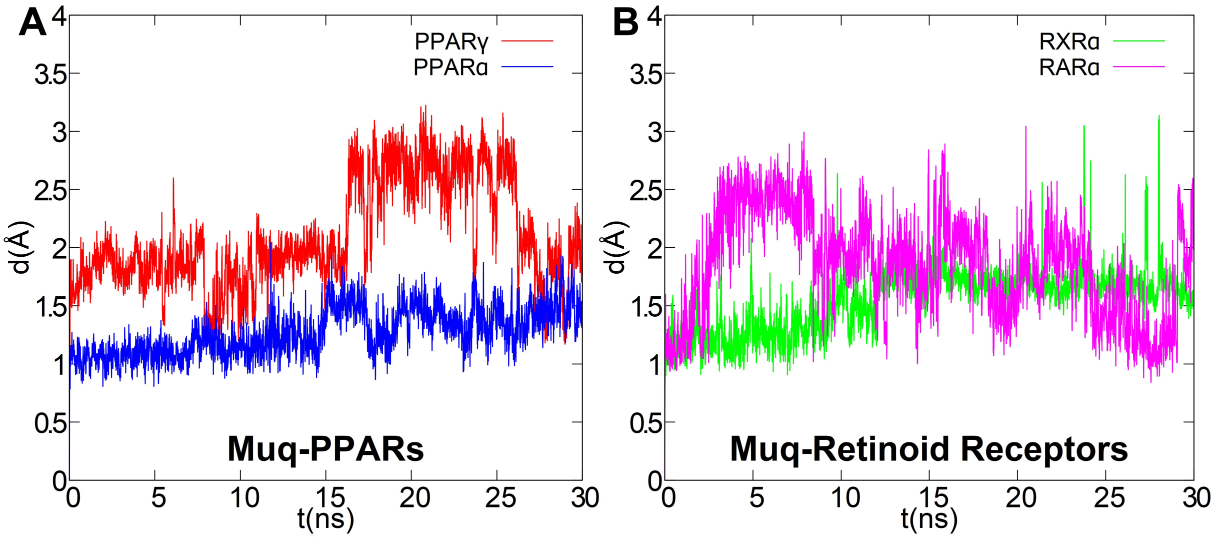

Figure S2: Evaluation of stability of the ligand binding mode during the last 30ns of MD. Rmsd plots of Muq in PPARs (A) and retinoids receptors (B) complexes after protein backbone best-fit.
